# Frailty, appendicular lean mass, osteoporosis and osteosarcopenia in peritoneal dialysis patients

**DOI:** 10.1007/s40620-022-01390-1

**Published:** 2022-07-11

**Authors:** Andrew Davenport

**Affiliations:** 1 Department of Renal Medicine, Royal Free Hospital, UCL, University College London Medical School, London, UK; 2 Department of Nephrology, Royal Free Hospital, UCL, University College London, Rowland Hill Street, London, NW3 2PF UK

**Keywords:** Peritoneal dialysis, DXA, Ethnicity, Osteoporosis, Osteopenia, Gender, Diabetes

## Abstract

**Introduction:**

The pattern of chronic kidney disease mineral bone disorder (CKD-MBD) is changing with increasing numbers of elderly patients now treated by dialysis. The risk of falls and bone fractures increases with frailty and sarcopenia. As such we wished to review the association between osteoporosis and frailty and loss of appendicular lean mass (ALM).

**Methods:**

Dual-energy X-ray absorptiometry (DXA) was used to measure lumbar spine and femoral neck bone mineral density (BMD) and body composition. Osteoporosis and osteopenia were defined according to *T* scores. ALM was indexed to height (ALMI). Frailty was classified using the clinical frailty scale (CFS).

**Results:**

DXA scans from 573 patients, 57.8% male, 36.8% diabetic, mean age 61.0 ± 15.8 years, with a median 6.0 (2–20) months of treatment with PD were reviewed. Forty-two (7.3%) were classified as clinically frail, 115 (20%) osteoporotic, and 198 (34.6%) ALMI meeting sarcopenic criteria, with 43% of osteoporotic patients being osteosarcopenic. In a multivariable model, femoral neck BMD was associated with weight, standardised β (St β) 0.29, *p* = 0.004, ALM St β 0.11, *p* = 0.03 and Black vs other ethnicities St β 0.19, *p* = 0.02, and negatively with age St β −0.24, *p* < 0.001, and frailty St β −2.1, *p* = 0.04. *Z* scores (adjusted for gender and age) were associated with ALMI (*r* = 0.18, *p* < 0.001).

**Discussion:**

Osteoporosis is increasing with the numbers of elderly dialysis patients. As frailty and sarcopenia increase with age, then the risk of falls and bone fractures increases with osteosarcopenia. Whether interventions with exercise and nutrition can improve bone heath remains to be determined.

**Graphical abstract:**

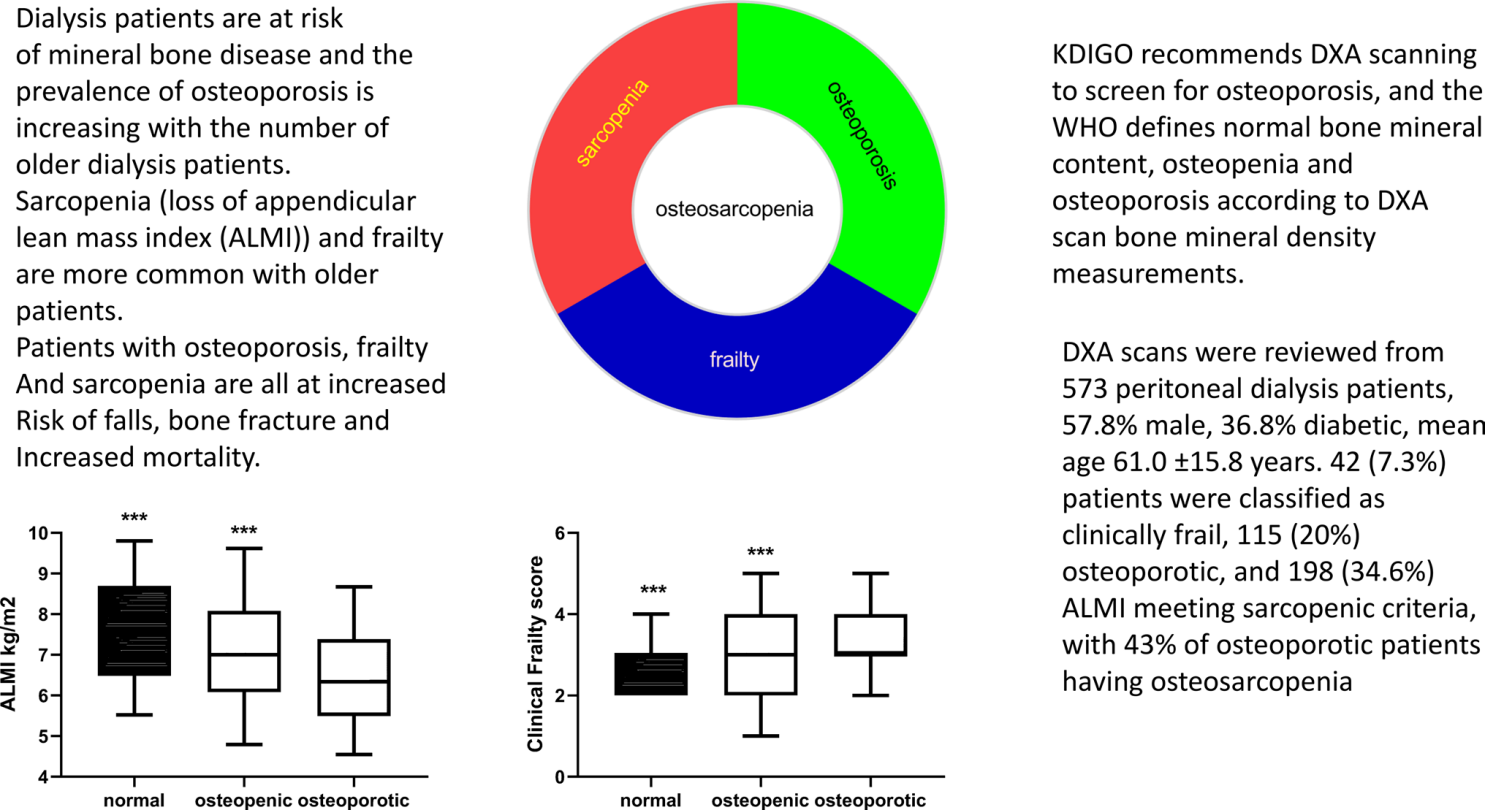

## Introduction

Patients with chronic kidney disease (CKD) are reported to have increased bone fracture rates compared with the general population [1]. In addition, CKD patients presenting with hip fractures are at increased risk of mortality, and hip fractures are also associated with substantial morbidity [1]. Whereas hip fracture rates are reported to be declining in the general population, studies from North America have suggested a temporal rise in the incidence of hip fractures in kidney dialysis patients, with the increase in fracture rate being associated with an increase in the number of older Caucasian dialysis patients [2].

Changes in the demographics of the dialysis population, with increasing numbers of older patients, lead to a more complex pattern of chronic kidney disease mineral bone disorder (CKD-MBD) due to varying amounts of osteoporosis and renal osteodystrophy [3, 4]. Muscle mass declines with age, but dialysis patients are at greater risk of muscle loss, more so than that which would be expected with age alone, often termed sarcopenia [5], and the combination with frailty [6] and reduced physical activity [7], leads to higher risks for both falls and bone fractures [8].

There have been debates as how to non-invasively investigate CKD-MBD in dialysis patients, but more recent studies have reported that bone mineral density (BMD) measured by dual energy X-ray absorptiometry (DXA) predicted fractures in dialysis patients, and as such the Kidney Disease Improving Global Outcomes (KDIGO) CKD-MBD group proposed that DXA scanning be introduced into clinical practice for dialysis patients [1]. The world health organization (WHO) and the International Society for Clinical Densitometry (ISCD) have issued guidelines defining osteopenia and osteoporosis based on BMD measured by DXA [9].

The United Kingdom (UK) introduced the 9-point Clinical Frailty Scale (CFS) into clinical practice as part of a holistic approach to patient care, with frailty defined as a score of greater than 4 [10]. As frail patients have an increased risk of osteoporosis [3], we wished to review the association between BMD, frailty, and reduced muscle mass in a cohort of peritoneal dialysis (PD) patients.

## Methods

We reviewed the DXA scans of a cohort of adult PD patients attending a UK university hospital. All scans were performed after drainage of PD dialysate, post voiding and with patients wearing only a thin gown (Hologic Discovery A (S/N87402.1), software version 13.5.2.1, Hologic, USA) [11]. Bone mineral density was measured at the lumbar spine (LS) (L1–L5) and femoral neck (FM), and additionally reported as *T*-scores, the bone density comparison to that of a 30-year-old healthy, gender-matched person and *Z* scores comparing bone density to the average values for a person of the same age and gender [12]. According to WHO criteria, patients were categorized into three groups: normal bone mineral density with a *T*-score no less than −1.0, osteopenia with a *T*-score between −1.0 and −2.5, and osteoporosis for patients with a *T*-score less than −2.5 [9, 12]. Appendicular lean mass (ALM) and body fat were also measured by DXA. ALM and body fat were indexed by height squared (ALMI) and as percentage of total body weight, respectively. Sarcopenia was defined by a reduced ALMI, according to male and female cut-off points as advised by the European Working Group on Sarcopenia in Older People 2 (EWGSOP2) [13], and the Asian Working Group for Sarcopenia [14], respectively.

Hospital computerised records were reviewed to retrieve patient demographics, relevant laboratory investigations and CFS scores [15]. Dietary protein intake was estimated by calculating normalized nitrogen appearance (nPNA) rates [16]. Patients were reviewed and provided with dietary advice by trained renal dietitians to avoid high phosphate foodstuffs. Standard clinical practice was to prescribe 20,000 IU of Vitamin D3 weekly to all patients.

### Statistical analysis

Normally distributed continuous variables were expressed by mean values ± standard deviation (SD), non-parametric continuous variables were reported as median (25th and 75th percentile). Categorical variables were expressed by frequencies and percentages. Standard analyses including *t*-test and Anova were used for parametric continuous variables, Mann–Whitney *U* test and Kruskal–Wallis for nonparametric continuous variables, and chi-square test was performed for categorical variables. Bonferroni and Games-Howell adjustments were made in cases of multiple testing. Univariate analysis was by Spearman correlation. Determinants of femoral neck bone mineral density were analysed using a step backward multivariable regression using variables associated with *p* < 0.1 on univariate analysis, and then variables were excluded if not statistically significant, unless they improved model fit. If required, nonparametric variables were log transformed. Analyses were performed using Statistical Package for Social Sciences (SPSS Version 27.0 software, IBM Corp., Armonk, New York, USA), Prism 9.4 (Graph Pad, San Diego, USA) and Microsoft Excel Version 2107 (Build 14228.20226). A two-tailed *p*-value < 0.05 was considered statistically significant.

### Ethics

This retrospective audit was conducted according to UK National Research Ethics guidelines and did not require additional local ethical approvals or individual patient consent. The audit was registered with the University hospital, and all data were anonymized.

## Results

DXA scans with measurements of ALM and FN BMD were available for review in 573 PD patients attending a university hospital PD program between 2004 and 2019; mean age 61.0 ± 15.8 years, 57.8% male, 36.8% with diabetes mellitus, who had been treated by PD for a median of 6 (2–20) months. Mean body mass index (BMI) was 26.3 ± 5.0 kg/m^2^, with an ALMI of 7.2 ± 2.3 kg/m^2^ and median percentage body fat of 30.2 (17.7–48.1) %. Patients were excluded if they had not had a DXA scan (awaiting living donor transplant, or transplanted before DXA scan appointment, unable to lie on DXA scanning table), limb amputee or paralysis, and if they did not have results of both LS and FN measurements (hip replacements), or patients did not attend for scanning.

Forty-two patients (7.3%) were classified as clinically frail with a CFS score of 5 or more, and patients were divided according to frailty (Table 1). These frail patients were older, with less ALM, more likely to have diabetes, and proportionally more White and Asian compared to those of Black ethnicity. Although LS BMD was lower, *T* and *Z* scores did not differ, whereas they were all lower at the FN.

**Table 1:** Patient demographics and body composition and bone densitometry measured by dual-energy X-ray absorptiometry (DXA)

Variable	All patients	Not frail	Frail
Number	573	531 (92.7)	42 (7.3)
Male (%)	331 (57.8)	311 (52.4)	20 (47.6)
Diabetic (%)	211 (36.8)	183 (33.3)	28 (66.7)***
Caucasian/Black/Asian %	49.2/22.9/27.9	45.3/88/66.7	54.7/12/33.3
Age years	61.0 ± 15.8	60.6 ± 16.0	70.4 ± 11.3***
PD treatment months	6.0 (2.0–20)	6.0 (2–29.5)	9 (3–20)
nPNA g/kg/day	0.92 ± 0.25	0.91 ± 0.29	0.84 ± 0.19
Body mass index kg/m^2^	26.3 ± 5.0	26.2 ± 4.8	26.5 ± 5.5
% Body fat	31.3 (22.2–38.2)	30.6 (22–38.2)	33.9 (30–41.1)
ALMI kg/m^2^	7.2 ± 2.3	7.3 ± 2.6	6.4 ± 1.6*
C reactive protein mg/L	4 (1–9)	4 (1–8)	6 (2–17)
PTH pmol/L	30.2 (17.7–48.7)	31.1 (17–50.6)	26.1 (15.6–28.3)*
LS BMD g/cm^2^	1.02 ± 0.21	1.03 ± 0.21	0.95 ± 0.21*
FN BMD g/cm^2^	0.71 ± 0.16	0.72 ± 0.15	0.61 ± 0.15***
*T* score LS	− 0.8 (− 1.9 to 0.2)	− 0.8 (− 1.8 to 0.4)	− 1.2 (− 2.4 to 0)
*T* score FN	− 1.7 (− 2.4 to 3.1)	− 1.6 (− 2.3 to − 0.9)	− 2.2 (− 3.0 to − 1.4)***
*Z* score LS	0.1 (− 1.0 to 1.4)	0.15 (− 1 to 1.4)	0.5 (− 1 to 1.85)
*Z* score FN	− 0.6 (− 1.1 to 0.1)	− 0.6 (–1.1 to 0.1)	− 0.8 (− 1.8 to − 0.2)**

Peritoneal dialysis (PD), normalised protein nitrogen appearance rate (nPNA), appendicular lean mass index (ALMI), C reactive protein (CRP), parathyroid hormone (PTH), activated vitamin D23 (alphacalcidol), elemental calcium in medications (calcium meds), lumbar spine (LS), femoral neck (FN), bone mineral density (BMD). Data expressed as integers, percentages, mean ± standard deviation and median (interquartile range). *T* and *Z* scores according to World Health Organization definitions [9]. Comparison vs Not frail *p < 0.05, **p < 0.01, ***p < 0.001

**Fig. 1:**
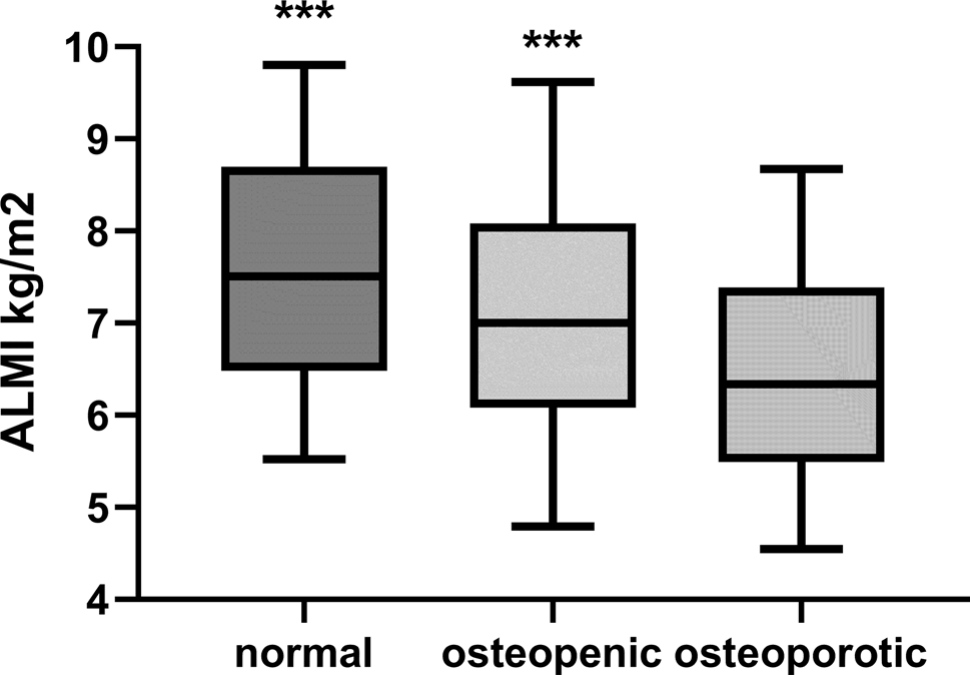
Appendicular lean mass index (ALMI) and World Health Organisation bone mineral density *T* scores at femoral neck as having normal, osteopenic and osteoporotic bone mineral density. ***p < 0.001 vs osteoporotic group

One hundred and ninety-eight patients (34.6%) had reduced muscle mass, and patients were then divided according to whether they had reduced ALMI, using the cut-off points for sarcopenia, as recommended by international guidelines (Table 2). Patients with ALMI values in keeping with sarcopenia were again more likely to be of Caucasian and Asian ethnicity compared to Black, and with greater CFS scores. BMI was not significantly different between the ethnic groups. Similarly, these patients with sarcopenic levels of ALMI had lower BMD, particularly at the FN (Fig. 1), with greater numbers of these patients classified as osteoporotic.

**Table 2:** Patient demographics and body composition and bone densitometry measured by dual-energy X-ray absorptiometry (DXA)

Variable	All patients	Normal ALMI	Low ALMI
Number	573	377 (65.8)	196 (34.2)
Male (%)	331 (57.8)	212 (56.2)	119 (60.7)
Diabetic (%)	211 (36.8)	134 (35.5)	77 (39.3)
Caucasian/Black/Asian %	49.2/22.9/27.9	55.6/85.7/60.2	44.4/14.3/39.8***
CFS score	3 (2–4)	3 (2–3)	3 (3–4)***
Age years	61.0 ± 15.8	60.0 ± 16.0	63.0 ± 16.0
PD treatment months	6.0 (2.0–20)	6.0 (2–22)	6.0 (2–18)
nPNA g/kg/day	0.92 ± 0.25	0.90 ± 0.25	0.93 ± 0.25
Body mass index kg/m^2^	26.3 ± 5.0	27.7 ± 4.9	23.8 ± 4.1
% Body fat	31.3 (22.2–38.2)	31.4 (22.4–39.0)	30.9 (21.6–36.4)
ALMI kg/m^2^	7.2 ± 2.3	8.0 ± 2.4	5.6 ± 0.9***
C reactive protein mg/L	4 (1–9)	4 (2–8)	4 (1–12)
PTH pmol/L	30.2 (17.7–48.7)	29.9 (18.1–49.8)	30.7 (21.6–36.4)
LS BMD g/cm^2^	1.02 ± 0.21	1.04 ± 0.21	0.98 ± 0.22**
FN BMD g/cm^2^	0.71 ± 0.16	0.74 ± 0.17	0.66 ± 0.13***
*T* score LS	− 0.8 (− 1.9 to 0.2)	− 0.7 (− 1.7 to 0.3)	− 1.1 (− 2.1 to 0.1)*
*T* score FN	− 1.7 (− 2.4 to 3.1)	− 1.55 (− 2.3 to − 0.9)	− 2 (− 2.5 to − 1.4)***
*Z* score LS	0.1 (− 1.0 to 1.4)	0.2 (− 0.9 to 1.5)	0 (− 1.2 to 1.2)
*Z* score FN	− 0.6 (− 1.1 to 0.1)	− 0.5 (− 1.1 to 0.1)	− 0.7 (− 1.3 to − 0.1)**

Peritoneal dialysis (PD), normalised protein nitrogen appearance rate (nPNA), appendicular lean mass index (ALMI), C reactive protein (CRP), parathyroid hormone (PTH), activated vitamin D23 (alphacalcidol), elemental calcium in medications (calcium meds), lumbar spine (LS), femoral neck (FN), bone mineral density (BMD). Data expressed as integers, percentages, mean ± standard deviation and median (interquartile range). *T* and *Z* scores according to World Health Organization definitions [9]. Comparison normal ALMI vs low ALMI (sarcopenic) *p < 0.05, **p < 0.01, ***p < 0.001

One hundred and fifteen patients (20%) were classified as osteoporotic based on femoral BMD. Almost half, i.e., 43% of the osteoporotic patients had sarcopenic levels of appendicular lean mass. The median PTH was 30.2 (17.7–48.1) pmol/L, serum calcium 2.32 ± 0.2 mmol/L, and median phosphate 1.35 (1.1–1.7) mmol/L. The median dose of activated vitamin D (alfacalcidol) prescribed was 0.5 (0.5–0.75) ug/week, along with an estimated elemental calcium content of phosphate binders of 0 (0–1500) mg/day, with patients routinely prescribed 20,000 IU of cholecalciferol weekly. There was no difference in PTH between those patients with normal femoral neck BMD, osteopenia and osteoporosis (median 31.1 (14.1–53.6) vs 29.9 (17.7–46.7) vs 26.5 (15.4–48.9) pmol/L), or active vitamin D prescription (median 0.5 (0.5–1.5) vs 0.5 (0.5–0.5) vs 0.5 (0.5–0.56) ug/week, respectively. However, patients with osteoporosis were prescribed less daily elemental calcium, as calcium-containing phosphate binders, compared to those with osteoporosis; (osteoporosis group median 0 (0–727) vs osteopenia 0 (0–1500) (*p* = 0.014) and osteoporosis 220 (0–1500) mg/day, (*p* = 0.01). There was no significant difference between the three main ethnic groups (White median 0 (0–1320), Asian 220 (0–1500) and Black 0 (0–1320) mg//day.

**Fig. 2:**
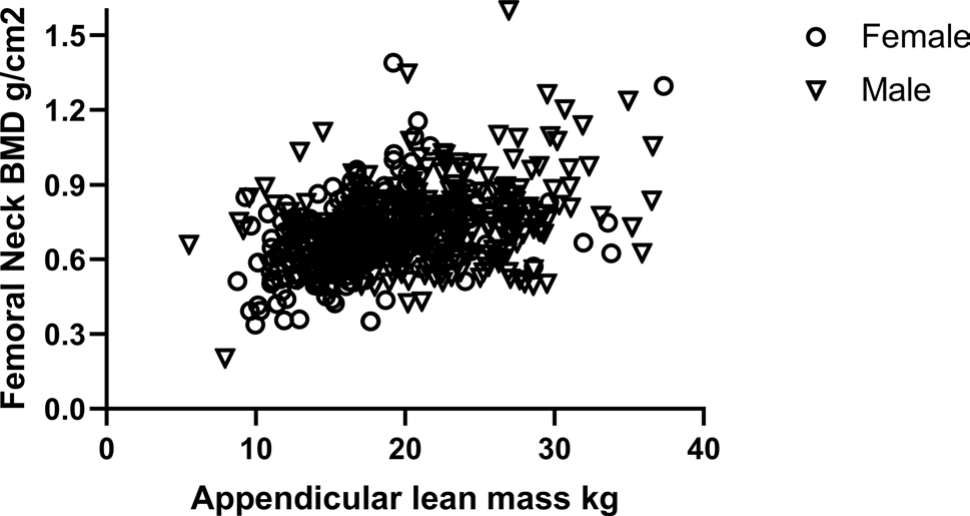
Spearman correlation and appendicular lean mass, Correlation for females *r* = 0.42, *p* < 0.001 and for males *r* = 0.30, *p* < 0.001

On univariate correlation, BMD at the FN was associated with increasing muscle mass (Fig. 2), weight, BMI, prescription of calcium-containing phosphate binders and male gender, and lower BMD was associated with increasing age, CFS score, and Caucasian and Asian ethnicity compared to Black and diabetes (Table 3).

**Table 3:** Spearman rho univariate association with femoral neck bone mineral density

Variable	Spearman rho	*p* value
Appendicular lean mass kg	0.38	< 0.001
Age years	0.37	< 0.001
Weight kg	0.37	< 0.001
Appendicular lean mass index kg	0.30	< 0.001
Body mass index kg/m^2^	0.27	< 0.001
Ethnicity	0.27	< 0.001
Clinical frailty score	− 0.25	< 0.001
Reduced appendicular lean mass	− 0.23	< 0.001
Daily elemental calcium medications mg	0.12	0.001
Months of peritoneal dialysis	− 0.11	0.003
Male gender	0.10	0.009
Diabetic	− 0.09	0.012

A multivariate step-backward regression model was developed with variables associated with BMD. Weight and ALM were independently associated with BMD, whereas age, frailty and Caucasian and Asian ethnicity were negatively associated with lower BMD (Table 4).

**Table 4:** Multivariable step backward model for variables independently associated with femoral neck bone mineral density

Variable	β	StE β	Std β	*T*	95% CL	*p* value
Weight kg	0.003	0.000	0.29	6.0	0.002–0.004	< 0.001
Age year	− 0.002	0.000	− 0.24	− 5.3	− 0.003 to − 0.002	< 0.001
Ethnicity	0.033	0.007	0.19	4.4	0.018–0.047	< 0.001
ALM kg	0.003	0.001	0.11	2.2	0.000–0.005	0.031
CFS	− 0.013	0.006	− 0.09	− 2.1	− 0.026 to − 0.001	0.041
Vintage	− 0.021	0.011	− 0.0.9	− 1.9	− 0.044 to 0.001	0.062

Ethnicity Black vs other races, appendicular lean mass (ALM), clinical frailty score (CFS), months of peritoneal dialysis treatment (vintage). Standard error beta (StEβ), standardised β (Std β), 95% confidence limits (95% CL). Model checked for collinearity (all > 0.7) and variable inflation factor (all < 1.2). Model *r*^2^ 0.314, adjusted *r*^2^ 0.304

## Discussion

The pattern of CKD-MBD has changed with the passage of time following the introduction of vitamin D analogues, withdrawal of aluminium-containing medications, introduction of non-calcium-containing phosphate binders, and the development of medical treatments for hyperparathyroidism, resulting in adynamic bone disease or low bone turnover now being the most common type of renal osteodystrophy [17].

We found no association between parathyroid hormone levels and BMD. Around 60% of our patents were classified as osteopenic, with 25% being osteoporotic, thus these patients would have an increased risk of bone fractures [18].

Osteoporosis can be non-invasively diagnosed according to BMD measurements made by DXA scanning [3, 18], and osteoporosis in CKD and dialysis patients increases the risk for both fractures and all-cause mortality [19]. Frailty and sarcopenia are also associated with osteoporosis and bone fracture [3], and it has been suggested that grades of frailty in the elderly could potentially assist in the assessment and management of osteoporosis [3]. As there are increasing numbers of elderly patients now being treated by dialysis, and 25% of our patients were aged 75 years or older, we wished to review the association between frailty, loss of muscle mass and osteoporosis in a cohort of peritoneal dialysis patients.

Using the CFS, only a minority of patients were classified as clinically frail [10]. These patients were older, with lower BMD, and correspondingly lower *T* and *Z* scores for the FN. Additionally, more diabetic patients were frail, and frail patients had a lower ALMI, Only around a quarter of our patients were aged 75 years or older, and this reflects the traditional practice in the UK that patients had to be capable of performing their own PD treatment at home, and it is only relatively recently that an assisted PD program has been introduced into clinical practice.

As frailty depends on muscle mass and function, we reviewed patients according to cut-offs for sarcopenic levels of ALMI [13, 14]. Patients with sarcopenic levels of ALMI had lower BMD, particularly at the FN with lower *T* and *Z* scores. There was an association between greater ALM and BMD at the FN with ALM decreasing progressively when comparing patients with normal BMD, osteopenia and osteoporosis.

In a multivariable model, BMD at the FN was associated with ALM, weight and Black ethnicity compared to those of Caucasian and Asian backgrounds, and negatively associated with increasing age, and CFS. Although longer treatment with PD was associated with lower BMD on univariate analysis, it was not statistically significant in the multivariable model.

We report an observational study demonstrating that although patients with CKD-MBD may potentially have a spectrum of bone disorders [1], the increasing number of older patients with loss of ALM and greater CFS scores leads to an increase in osteoporosis. As such, dialysis patients also demonstrate an overlap between osteoporosis and sarcopenia, which has recently been termed osteosarcopenia [20]. In our cohort, 43% of patients with sarcopenic levels of ALMI were classified as osteoporotic. The aetiology of osteosarcopenia is thought to be multifactorial with several factors linking muscle and bone function, including genetics, age, inflammation and obesity [20]. In keeping with our data, showing a difference in BMD between ethnic groups, previous studies have also demonstrated a difference in body composition [21], and the prevalence of osteoporosis between patients of Black ethnicities compared to those of Caucasian and Asian backgrounds [22].

Similarly, although there was an association with body weight and BMD, we did not find any association with BMI or percentage body fat. This may be due to differences between CKD patients and the general population, as dialysis patients may lose ALM but gain fat weight, sometimes termed sarcopenic obesity [23]. When we reviewed patients who had sarcopenic levels of ALM, we found that these patients did have a lower BMI compared to those with normal levels of ALM, but not statistically lower. Although it has been suggested that inflammation increases osteosarcopenia, we did not note any association between C reactive protein, a marker of inflammation and BMD. Others have suggested that osteoporosis and sarcopenia are linked to nutritional deficits in geriatric inpatients [24]. However, we did not find any association between estimated dietary protein nitrogen appearance rates, calculated from spent dialysate and urine collections, and either BMD or ALM. This may be due to the dietary restrictions imposed on dialysis patients to limit sodium and phosphate intake, coupled with reduced physical activity [25, 26]. In addition, patients with progressive chronic kidney disease often naturally restrict dietary protein, and although protein intake may be encouraged once starting dialysis, appetite may be suppressed in PD patients due to the presence of intra-abdominal fluid, increased reflux oesophagitis and glucose absorption from the peritoneal dialysate [27, 28]. Moreover, we studied a multi-ethnic group of patients, and as such, ethnicity may have also influenced dietary intake. Centre practice was to preferentially prescribe non-calcium-containing phosphate binders due to concerns of vascular calcification. Whereas there were no differences in the prescription of native or activated forms of vitamin D, osteoporotic patients were prescribed less elemental calcium in phosphate binders. Although associated with femoral neck BMD, elemental calcium prescription was not retained in the multivariable model.

We report an observational study which demonstrates an association between reduced BMD and ALM. Similarly, when using WHO grading, patients with osteoporosis had lower ALM, both for *T* scores, adjusted for gender, and also *Z* scores, when additionally adjusted for age [9]. However, we are unable to adjudge causality, and although there was a suggestion that the reduction in BMD was greater with longer duration of dialysis treatment, we were unable to confirm this in a cross-sectional study. We did not record dietary histories, and dietary intake in dialysis patients does vary between ethnic groups and so we are unable to comment on dietary calcium intake [29]. Although Asian patients were prescribed marginally more calcium in phosphate binders, we have no compliance data, and different foodstuffs can affect the bioavailability of both calcium and phosphate, so for example Asian patients are more likely to eat greater amounts of phytates in their diet, thereby affecting calcium bioavailability. Similarly, we did not record physical activity, which is linked to active energy expenditure and muscle mass [30, 31]. On the other hand, the strength of our study is the very large number of PD patients included, in particular the ethnic diversity. Secondly, we report on femoral neck BMD, whereas whole body and lumbar spine measurements can be confounded by differences in skull calcification between genders [19], overlying vascular calcification, implantable cardiac and other devices, vertebral crush fractures, and electron dense material in the bowel, such as lanthanum-containing medications [32].

Our study demonstrates that PD patients are at risk of osteosarcopenia. As falls are more common in patients with sarcopenia, and equally common in PD and haemodialysis patients, then osteoporotic PD patients are also at risk of increased mortality from fractures. The pattern of CKD-MBD is changing over time, with increasing prevalence of osteopenia and osteoporosis. As more and more older and frail patients are now offered treatment by PD in Europe and North America, this will require a change in clinical practice to develop treatments designed to reduce the loss of calcium from bones and improve bone structure. Patients with osteoporosis had lower ALM, and therefore, whether osteosarcopenic PD patients will benefit from non-pharmaceutical treatments such as exercise, and if nutritional programs will improve CKD-BMD and muscle mass remains to be determined.

## Data Availability

Data held UCL Department of Nephrology V drive, data availability upon reasonable request and within NHS guidelines.
